# Therapy-Related Toxicity in Bladder Cancer Treatment: A Case Report

**DOI:** 10.7759/cureus.91905

**Published:** 2025-09-09

**Authors:** José Miguel Rocha, Ricardo Fernandes, Luísa Queiros, Jorge Rodrigues, Diana Freitas

**Affiliations:** 1 Medical Oncology, Unidade Local de Saúde de Braga, Braga, PRT

**Keywords:** autoimmune disorders, bladder cancer, cancer toxicity detection, immune-related adverse events, immunotherapy, targeted therapy, urothelial carcinoma

## Abstract

Bladder cancer is a commonly diagnosed cancer worldwide. Within muscle-invasive bladder cancer (MIBC), the disease may present as localized or as metastatic. MIBC requires distinct therapeutic approaches, ranging from local treatments for preventing recurrence to systemic therapy. In this case report, we present the clinical case of a 67-year-old man diagnosed with muscle-invasive urothelial bladder cancer. His treatment through different stages of the disease is described, with the inclusion of immunotherapy-related toxicity. This case illustrates the complex treatment landscape for advanced MIBC, for which curative-intended strategies are standard, but disease relapse frequently occurs. The relevance of individualized treatment is highlighted. Advances in immunotherapy and the use of targeted therapy and antibody-drug conjugates provide hope for improved outcomes, but careful and often multidisciplinary management of treatment-related complications remains critical for optimizing patient care.

## Introduction

Bladder cancer is the ninth most frequently diagnosed cancer worldwide, with 614,000 new cases and 220,000 deaths estimated to have occurred in 2022 [1]. Bladder cancer can be classified into two main categories: non-muscle-invasive bladder cancer (NMIBC) and muscle-invasive bladder cancer (MIBC). Within MIBC, the disease may present as localized or as metastatic. Patients with localized disease require more intensive treatment, which often involves a combination of systemic therapy (chemotherapy, immunotherapy) and surgery and typically requires a multidisciplinary approach. In metastatic bladder cancer, the focus lies in extending the patient's lifespan and improving or maintaining their quality of life. A variety of drugs with different mechanisms are used in this stage. Approximately 5% of patients have metastatic disease at the time of diagnosis, which is associated with an unfavorable prognosis [2].

In this report, we describe a case of a patient diagnosed with urothelial bladder cancer and his journey through the various phases of the disease. The description includes information on pembrolizumab-related musculoskeletal toxicity and its management.

## Case presentation

We describe the case of a 67-year-old man with a past medical history of second-degree atrioventricular block. He had an implanted pacemaker, dyslipidemia, and a non-specified osteoarticular disease, and he was an active smoker (17 pack-years). No relevant family background and no allergies were reported.

He presented at our center with gross hematuria. Abdominal and pelvic ultrasound showed a 7-cm mass on the right wall of the bladder. A transurethral resection of the bladder was performed. The pathology report revealed a high-grade papillary urothelial carcinoma staged as pT2. A staging thoracic-abdominal-pelvic computed tomography (CT) scan revealed an abnormally high number of nonspecific retroperitoneal and lumbar aortic lymph nodes and a 9-mm right common iliac adenomegaly.

In a multidisciplinary team meeting, neoadjuvant chemotherapy consisting of four cycles of cisplatin (70 mg/m^2^) and gemcitabine (1,000 mg/m^2^) was proposed for the patient, starting in June and ending in September 2021. The reevaluation CT scan showed a partial response to treatment, and the patient was submitted to radical cystectomy with Bricker urostomy. The pathology report confirmed response to treatment (ypT1N0), and ongoing monitoring was decided.

**Figure 1:**
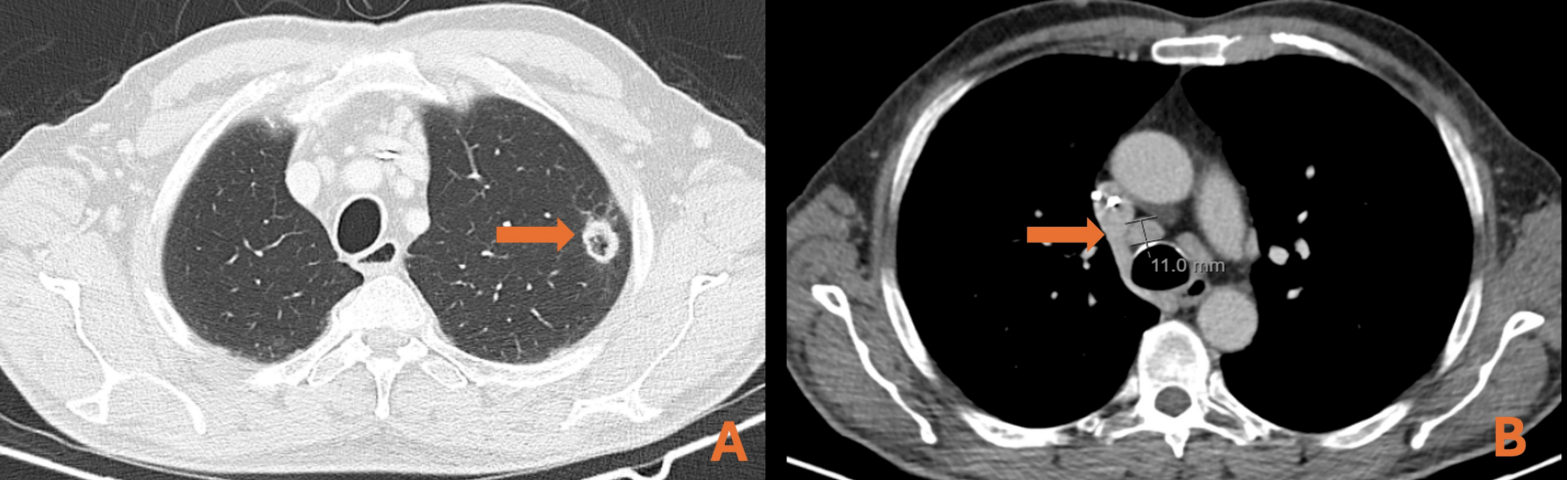
CT of the chest, abdomen, and pelvic showing (A) the pulmonary lesion and (B) the de novo nodes CT: computed tomography

Four months after surgery, follow-up CT showed a 24-mm pulmonary lesion on the superior left lobe and the mesenteric, lumbar aortic, and external iliac nodes, all de novo (Figure 1).

Lung lesion biopsy was performed, and the pathology report confirmed a urothelial carcinoma metastasis. Initiating first-line therapy with pembrolizumab was proposed for the patient, given the disease progression under 12 months of platinum-based therapy.

The first cycle of pembrolizumab (200 mg) was initiated in August 2022. After the first cycle, the patient developed a grade 2 (Common Terminology Criteria for Adverse Events (CTCAE) v5.0) migratory arthralgia involving his shoulders and small joints of his hands and feet, edema of the phalangeal joints in his hands, and pelvic and shoulder blade stiffness, with functional limitations on daily activities. The patient was medicated with prednisolone 10 mg/day with slight clinical improvement. After the second cycle of immunotherapy, the patient experienced worsening of the musculoskeletal symptoms.

The treatment was discontinued, and the patient was referred to the Rheumatology department. Blood analysis showed elevated creatine kinase and myoglobin levels and positive results for anti-SSA and Jo-1. Rheumatoid factor and anti-dsDNA tests were negative, and the erythrocyte sedimentation rate was normal. A high-resolution chest CT suggested pulmonary interstitial involvement with diffuse ground-glass opacities.

Following these findings, the patient was diagnosed with Jo-1-positive antisynthetase syndrome, and he began therapy with subcutaneous methotrexate 15 mg/week while maintaining prednisolone 10 mg/day with clinical improvement.

Suspension of immunotherapy was decided, since active autoimmune disease was present. Since the patient maintained a good performance status (Eastern Cooperative Oncology Group (ECOG) Performance Status (PS) 1), second-line therapy with enfortumab vedotin (EV) (1.25 mg/kg) was proposed.

**Figure 2:**
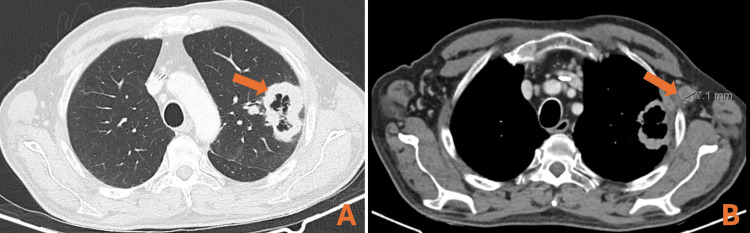
Restaging CT scan showing (A) increased lung lesion and the development of (B) left axillary lymph node CT: computed tomography

CT of the chest, abdomen, and pelvis was performed for restaging, showing the dimensional increase of the pulmonary lesion (6×4 cm) and a de novo left axillary lymph node (17 cm) (Figure 2).

The patient completed the first cycle of EV in December 2022. Disease response evaluation by CT was performed after three months of treatment, showing stable disease.

The antibody-drug conjugate treatment was well tolerated, with a single episode of a self-limited grade 1 rash on days 2 and 3 after the first cycle was reported. No other toxicities were reported. Of note, a 14-day hospitalization in the Infectiology department occurred due to *Listeria monocytogenes* bacteremia without an evident focus. Central nervous system infection and endocarditis were excluded, and the patient was discharged after a course of targeted antibiotic therapy.

**Figure 3:**
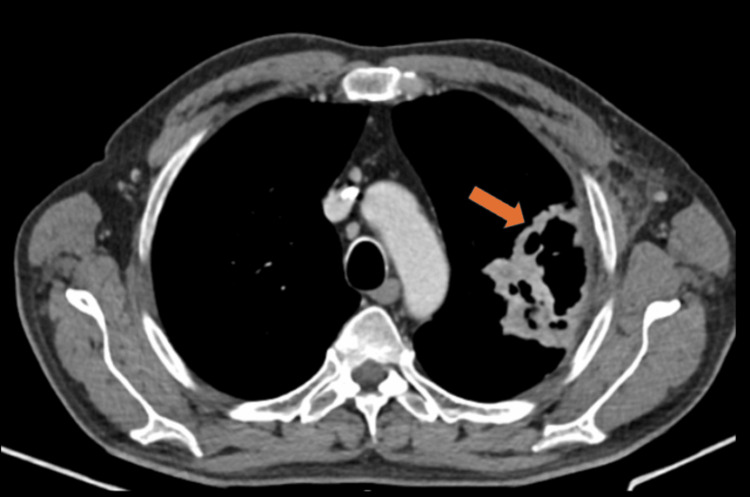
CT scan showing the dimensional increase of the lung lesion, indicating disease progression CT: computed tomography

The patient was under treatment with EV for a total of nine cycles. In September 2023, the CT showed disease progression with an increase of the lung lesion and of the left axillary node (Figure 3).

Third-line weekly paclitaxel was proposed in light of disease progression and the good overall performance status of the patient. As the patient was entering the later lines of systemic therapies available, a genomic test by next-generation sequencing (NGS) was performed to identify potential targetable mutations.

**Figure 4:**
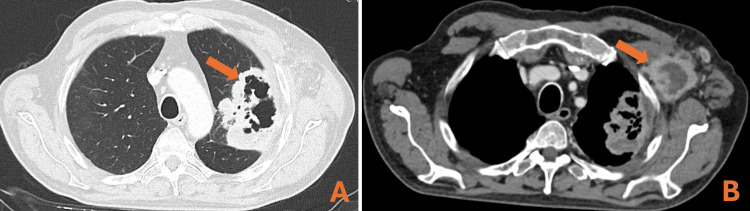
Response to treatment evaluation by CT scan demonstrating the increment of the lung lesion (A) and left axillary node (B) CT: computed tomography

The patient completed 13 cycles of weekly paclitaxel (80 mg/m^2^) with good tolerance until February 2024 when CT demonstrated disease progression (Figure 4). Results from the NGS were available, and a FGFR3 mutation (S249C) was found.

Since the patient kept an adequate performance status, fibroblast growth factor receptor (FGFR)-directed therapy with erdafitinib was proposed. The patient started therapy with this agent in April 2024, with involvement of the Ophthalmology department as required for this class of agents. After three months of treatment, the patient developed right retinal detachment and grade 2 nail loss, leading to drug discontinuation. After clinical resolution with conservative measures, the patient resumed treatment with dose reduction. In November 2024, follow-up CT scans revealed disease progression in the lungs, bone, and axillary lymph node.

The patient was referred for best supportive care and eventually passed away the following month.

## Discussion

The case described in this report included various stages of advanced MIBC, with setbacks and complications that should ideally be approached in a multidisciplinary context.

The majority of patients with MIBC present with primary invasive bladder cancer, and around 15% have a previous history of NMIBC [2]. In the absence of metastatic disease, the standard treatment for these patients is cisplatin-based neoadjuvant chemotherapy followed by radical cystectomy with bilateral pelvic lymphadenectomy. Cystectomy alone is associated with five-year survival in 50% of the patients [3], whereas the addition of cisplatin-based neoadjuvant chemotherapy has demonstrated a significant survival benefit with a 5% absolute improvement in five-year overall survival and a 9% absolute increase in five-year disease-free survival in comparison with surgery alone [4]. Cisplatin plus gemcitabine or accelerated methotrexate, vinblastine, adriamycin, and cisplatin are the chemotherapy regimens of choice in this setting. Nevertheless, around half the patients relapse, with local recurrence accounting for up to one-third of the cases, as illustrated in the present case, whereas the development of distant metastatic lesions is more common [5].

Since the standard treatment with cisplatin-based combinations was established, first-line treatment of advanced or metastatic urothelial cancer remained unchanged for around 20 years. Maintenance treatment with avelumab for patients with locally advanced or metastatic disease who were progression-free following platinum-based chemotherapy showed a survival benefit in comparison with the best supportive care [6]. Results from two randomized clinical trials enabled establishing a new standard of care in this setting, as both demonstrated a survival benefit against platinum-based chemotherapy [7,8]. Currently, EV-pembrolizumab is the treatment of choice in the first-line setting, regardless of eligibility for platinum-based therapy. Platinum-based combinations with the addition of nivolumab are also an option in these patients.

With this evolving scenario, optimal treatment selection in the second and further lines becomes a challenge. For tumors that have relapsed or progressed after platinum-based therapy and were not exposed to immunotherapy, pembrolizumab demonstrated an overall survival advantage compared with other chemotherapy agents (10.3 vs. 7.4 months; hazard ratio 0.73; 95% CI: 0.59-0.91), with a favorable toxicity profile [9]. Immunotherapy agents, such as immune checkpoint inhibitors (ICIs), have a particular toxicity profile derived from their mechanism of action inducing immune system activity.

The primary function of immune checkpoints is to regulate the immune response, preventing excessive activation and promoting self-tolerance by deactivating cytotoxic T cells. Normal cells utilize these checkpoints to protect healthy tissues by preventing immune-related damage. However, certain cancer cells take advantage of these mechanisms by engaging with receptors on cytotoxic T cells, allowing them to escape detection and suppression by the immune system [10].

ICIs work by targeting key molecules such as cytotoxic T-lymphocyte antigen 4 (CTLA-4) and programmed death receptor 1 (PD-1) and its ligand, programmed death-ligand 1 (PD-L1), which are often found in high levels within certain tumor microenvironments. This overexpression allows tumors to evade cytotoxic T cells, and blocking these pathways helps restore the immune system's ability to recognize and target cancer cells [10,11]. The infiltration of healthy tissues by these improperly regulated T cells is the probable cause of toxicity, with organ-specific inflammation and damage, although an increased production of antibodies has been described [11,12].

Up to 85% of patients experience immune-related adverse events following treatment with anti-CTLA-4 agents such as ipilimumab, while up to 70% report such events after receiving inhibitors of the PD-1 pathway. Combination ICI therapy exposes patients to a higher incidence of toxicity and organ-specific adverse events, which vary according to the specific ICI agent. Generally, colitis and hypophysitis are associated with anti-CTLA-4 agents, while pneumonitis and thyroid dysfunction are more common with anti-PD-1 antibodies [11]. Rheumatologic symptoms have also been associated with ICI therapy, with reports indicating a prevalence of up to 40% for arthralgia and 20% for myalgia in affected patients. These complications are more common with anti-PD-1 antibodies, and clinicians should remain highly vigilant for inflammatory arthritis and ensure timely referral to a rheumatologist, as irreversible erosive joint damage can occur rapidly. Although prednisone is the standard treatment, steroid-resistant cases have been managed with disease-modifying anti-rheumatic drugs or TNF-alpha inhibitors [11,13]. Physicians should also be particularly cautious when patients have pre-existing or unknown autoimmune conditions, as this population has been historically excluded from clinical trials with ICI therapy, resulting in limited real-world data. In these patients, the risk of developing a flare of the underlying disease or a de novo immune-related adverse event goes up to 50%. In this population, optimizing immunosuppression, with a goal of <10 mg of prednisone daily prior to ICI initiation, is recommended [12,14].

Therefore, appropriate supervision and early detection of immune-related events are of great importance to enable optimal benefits from treatment by ensuring its safety. In the described case, we witnessed a reactivation of an unknown autoimmune syndrome after the introduction of pembrolizumab. The diagnosis was possible owing to a multidisciplinary approach, with the findings from clinical and imaging work-up meeting the diagnostic criteria for antisynthetase syndrome. These criteria included the presence of the respective antibody, interstitial lung disease not attributable to another cause, and musculoskeletal signs [15].

In patients previously exposed to platinum-based chemotherapy and immunotherapy, other agents have demonstrated positive results. EV is a nectin-4-directed antibody drug conjugate that has been evaluated in phase 2 and phase 3 trials in advanced or metastatic urothelial cancer with progression after chemotherapy and checkpoint inhibition. The phase 3 trial showed response rate, progression-free survival, and overall survival favoring EV compared with chemotherapy agents, such as vinflunine and taxanes [16].

Genomic profiling of urothelial cancer has revealed targetable genomic alterations, including those in the gene encoding FGFR. Alterations in this gene are observed in around 20% of advanced or metastatic urothelial cancers [17]. Erdafitinib is an oral selective pan-FGFR tyrosine kinase inhibitor. The phase 3 THOR trial cohort 1 compared erdafitinib with chemotherapy (docetaxel or vinflunine) in patients with the aforementioned genomic alterations after progression after one or two prior treatments, with at least one including an ICI. The results showed an overall survival and progression-free survival benefit favoring erdafitinib (12.1 vs. 7.8 months (p=0.005) and 5.6 vs. 2.7 months (p<0.001), respectively), after previous anti-PD-1 or anti-PD-L1 treatment. Treatment-related toxicity grade ≥3 was similar in the two groups, and in the erdafitinib group, the most common adverse events of grade 3 or higher were palmar-plantar erythrodysesthesia syndrome (9.6%), stomatitis (8.1%), onycholysis (5.9%), and hyperphosphatemia (5.2%) [18]. Based on findings in the latter trial, erdafitinib was approved in patients harboring susceptible FGFR3 genetic alterations who have previously received at least one line of therapy containing a PD-1 or PD-L1 inhibitor in the unresectable or metastatic disease setting.

Other chemotherapy agents, such as taxanes, are a poorer alternative in comparison with EV or erdafitinib in tumors that progressed on platinum combinations and checkpoint inhibition, with response rates of approximately 20% [19]. Studies such as an ongoing phase 1b trial of nivolumab for patients with autoimmune disease and advanced cancer [20] may provide support for the development of potential treatment strategies to optimize the use of ICI therapies in a wider population of cancer patients.

## Conclusions

This case highlights the need for the careful monitoring and proactive management of immune-related adverse events in patients treated with ICIs. Multidisciplinary approaches and ongoing research are essential to balance therapeutic benefits and immunological risks, especially in patients with undiagnosed autoimmune diseases.

The advances observed in recent years, with the development of conjugated antibodies, immunotherapy, and targeted therapies, have expanded the range of therapeutic options for urothelial cancer patients. This expansion enables a tailored and personalized approach for each case, taking into account the patient's profile, functional status, comorbidities, and preferences. Treatment selection and sequencing, mainly in the second and subsequent lines, depend on which drugs are available in each center and which therapy was given in the first line. As data on these latter lines are highly variable, clinical trial enrollment should be considered.
